# HOUSING CONDITIONS, SOCIAL CAPITAL, AND SUBJECTIVE WELL-BEING AMONG MIGRANT OLDER ADULTS IN INDIA

**DOI:** 10.1093/geroni/igac059.2424

**Published:** 2022-12-20

**Authors:** Sruthi Anilkumar Hemalatha, Nawaj Sarif

**Affiliations:** International Institute for Population Sciences, Mumbai, Maharashtra, India; International Institute for Population Sciences, Mumbai, Maharashtra, India

## Abstract

Aging and migration are complex multidimensional processes affected by various factors at different levels. According to the most recent Census of India (2011), more than half of the older adults in India are migrants. Migration at an older age is considered risky and vulnerable because it may lead to many problems. Changes in the environment and living place at an older age may disconnect their social network and affect their health. Therefore, this study examines the housing conditions, social networks, and subjective well-being of older adult migrants in India using data from Longitudinal Aging Study in India, Wave 1 (2017-18). The bivariate and multivariate analyses show significant socio-economic differences in living arrangements and access to better housing facilities among older adult migrants. Additionally, there exist state-wide differences in the social capital of older migrant adults. The likelihood of having poor social capital is higher among migrant older adults than non-migrant older adults. It also reveals that the subjective well-being of older adult migrants is strongly associated with their housing conditions, social networks, and other socio-economic factors. Thus, the paper finds environmental factors and social support essential for better health and well-being, especially for older adults, especially those in new surroundings. Therefore, extensive research is needed to understand the situation of migrant older adults in India to formulate appropriate policies for the same.

